# Connecting the molecular dots from inaccessible antigen to organ injury in the pathogenesis of cardiac manifestations of neonatal lupus

**DOI:** 10.1186/ar3975

**Published:** 2012-09-27

**Authors:** JP Buyon

**Affiliations:** 1 New York University School of Medicine, New York, NY, USA

## 

Only 2% of fetuses exposed to anti-SSA/Ro antibodies have cardiac manifestations of neonatal lupus (cardiac NL, referred to herein as congenital heart block), yet these antibodies are present in >85% of mothers whose fetuses are identified with conduction abnormalities in a structurally normal heart. This disparity implies that the antibodies are necessary but insufficient to cause disease, and that the final pathway to fibrosis may be variable: kept totally in check in most fetuses (normal sinus rhythm), subclinical in others (first-degree block) and fully executed in very few (advanced block). A further challenge to defining the pathology of disease is the intracellular location of the target antigens, thus raising questions regarding accessibility to maternal antibodies. Our research has incorporated three components in the presumed cascade from antibody to tissue injury: apoptosis to provide the antigenic complex; macrophage uptake and secretion to drive inflammation and scarring; and persistence of the myofibroblast to drive replacement of healthy cardiac tissue. The totality of experimental data in the last 2 years supports that the watershed may involve conformational properties of Ro60 that influence its surface translocation to an apoptotic cardiocyte surface, its capacity to bind Y RNA and its effect on the biology of the urokinase plasminogen activator (uPA)/urokinase plasminogen activator receptor (uPAR) system. By exploiting murine fibroblast cell lines transfected with Ro60 modified to alter RNA binding sites, very recent data suggest that ssRNA (probably hY3 RNA) is required for surface translocation during apoptosis. However, the accessibility of Ro60 to cognate maternal antibody may be influenced by additional factors. In this regard, Ro60 expressed on the surface of an apoptotic cardiocyte binds domain V of β2GPI with high affinity and effectively blocks binding of anti-Ro60 antibodies. In an important translational step to humans, significantly lower levels of circulating β2GPI are associated with disease as assessed with umbilical cord blood comparing affected and unaffected fetuses. The importance of intact β2GPI as a protective factor became increasingly evident as we continued our search for the molecular explanation of a critical observation that anti-Ro60 antibodies inhibit the uptake of apoptotic cardiocytes by healthy cardiocytes. Although a counter-receptor for Ro60 on the healthy cardiocyte was considered, experiments revealed that anti-Ro60 binding to Ro60 (opsonization, formation of immune complexes) is associated with a conformational change of uPAR resulting in two functional consequences, one of which is a 'don't eat me' signal to healthy cardiocytes. The other is uPA activation leading to proteolytic cleavage of plasminogen and the generation of plasmin. The latter cleaves β2GPI, which renders it incapable of binding to Ro60 thus favoring the continued formation of immune complexes on the apoptotic surface. Moreover, the generation of plasmin also promotes the activation of TGF-β. Accordingly, the uPAR/uPA system plays a profibrotic role in the early and expansive phases of disease and the levels of uPAR, uPA, and plasminogen are increased in affected fetuses compared with healthy siblings. The potential of ssRNA to perpetuate an inflammatory step via a Toll-like receptor (TLR)7 pathway has been experimentally substantiated by the transdifferentiation of human fetal cardiac fibroblasts to a scarring phenotype following exposure to supernatants generated by incubation of macrophages with surrogate immune complexes comprised of Ro60, hY3 and affinity-purified anti-Ro60 antibody. Further evidence for a macrophage contribution to injury was provided by immunohistochemistry, which demonstrated not only a macrophage infiltrate in three hearts from anti-Ro-exposed fetuses dying with cardiac NL, but the formation of multinucleated giant cells. With regard to the fibroblast, we posit that these cells are themselves a major source of TGF-β and that endothelin-1 is one of the components responsible for the profibrosing effects generated by TLR signaling in the macrophages. The potential pathologic role of TLR signaling prompted the initiation of a case-control study in which we demonstrated that the use of hydroxychloroqine, which inhibits endosomal acidification, decreases the risk of cardiac NL. A prospective study to prevent recurrent disease has just been initiated.

